# Mitochondrial Transplantation Enhances Phagocytic Function and Decreases Lipid Accumulation in Foam Cell Macrophages

**DOI:** 10.3390/biomedicines10020329

**Published:** 2022-01-30

**Authors:** Soraya Játiva, Priscila Calle, Selene Torrico, Ángeles Muñoz, Miriam García, Ivet Martinez, Anna Sola, Georgina Hotter

**Affiliations:** 1 M2rlab-XCELL, 28010 Madrid, Spain; sjativa@m2rlab.com (S.J.); pcalle@m2rlab.com (P.C.); storrico@m2rlab.com (S.T.); mgarcia@m2rlab.com (M.G.); 2 Facultat de Medicina, Universitat de Barcelona, 08036 Barcelona, Spain; 3 Department of Experimental Pathology, Instituto de Investigaciones Biomédicas de Barcelona-Consejo Su Perior de Investigaciones Científicas Institut d’Investigacions Biomèdiques August Pi i Sunyer (IIBB-CSIC-IDIBAPS), 08036 Barcelona, Spain; angeles.munoz@iibb.csic.es (Á.M.); ivet.martinez.anguita@gmail.com (I.M.); 4 Department of Experimental Nephrology, Institut d’Investigació Biomèdica de Bellvitge (IDIBELL), Hospitalet de Llobregat, 08908 Barcelona, Spain; asola@idibell.cat; 5 CIBER-BBN, Networking Center on Bioengineering, Biomaterials and Nanomedicine, 50018 Zaragoza, Spain

**Keywords:** macrophage, foam cell, 7-ketocholesterol, CPT1a, phagocytosis, inflammation

## Abstract

Macrophages have mechanisms for eliminating cholesterol from cells. If excess cholesterol is not eliminated from the macrophages, then transformation into a foam cell may occur. Foam cells are a hallmark of the atherosclerotic lesions that contribute to the development and rupture of atherosclerotic plaques. Several in vitro and in vivo studies have shown changes in the macrophage phenotype and improved phagocytosis after the acquisition of functional mitochondria. However, the effect of mitochondrial transplantation on promoting phagocytosis and phenotypic changes in lipid-loaded macrophages leading to foam cells has not been studied. We aimed to prove that the transplantation of healthy mitochondria to highly cholesterol-loaded macrophages induces macrophage phagocytosis and reduces the macrophage shift towards foam cells. For this purpose, using a murine macrophage cell line, RAW264.7, we determined if mitochondria transplantation to 7-ketocholesterol (7-KC)-loaded macrophages reduced lipid accumulation and modified their phagocytic function. We evidenced that mitochondrial transplantation to 7-KC-loaded macrophages reestablished phagocytosis and reduced lipid content. In addition, CPT1a expression and anti-inflammatory cytokines were restored after mitochondrial transplantation. We have developed a potential therapeutic approach to restore foam cell functionality.

## 1. Introduction

It is known that mitochondrial intercellular transfer promotes the integration of mitochondria into the endogenous mitochondrial network of recipient cells, contributing to changes in their bioenergetics status and other functional skills, not only in vitro but also in vivo [1]. In addition, several in vitro and in vivo studies have shown that mitochondria transported from mesenchymal stem cells (MSCs) to macrophages can induce the selective differentiation of macrophages towards the anti-inflammatory M2 phenotype and can contribute to the antimicrobial effect of MSCs [2,3]. In acute respiratory distress syndrome, oxidative phosphorylation (OXPHOS) activity and the phagocytosis of macrophages were stimulated after they acquired functional mitochondria from the MSCs [3,4], and it has been hypothesized that stimulated OXPHOS was responsible for the macrophage conversion to the M2 phenotype [3]. In turn, the inhibition of intercellular mitochondrial transfer by affecting the mitochondria of MSCs [3] or by blocking the transfer pathway [3,4] reduces the phagocytosis and bioenergetics of the macrophages. Thus, these results suggest that mitochondrial transplantation could be considered to be a method to restore a compromised phagocytosis.

Foam cells are a hallmark of the atherosclerotic lesion, contributing to the development and rupture of atherosclerotic plaques [5,6,7]. Moreover, the formation of foam cells is accompanied by an inflammatory reaction, and it is known that lipid load macrophages induce NOD-like receptor pyrin domain-containing-3 (NLRP3) inflammasome activation [8,9]. In other experimental models in the spinal cord, it has been proven that intracellular lipid load modifies macrophage phagocytosis and inflammasome activation. This lipid load is due to the high cholesterol content of myelin debris, leading to deficient phagocytosis in the macrophages of the spinal cord [10]. The lipid-laden macrophages are no longer able to engulf waste material, as their phagocytic activity is reduced, perpetuating inflammation and inflammasome activation. Consequently, these findings suggest that the high intracellular cholesterol content in macrophages results in NLRP3 activation and phagocytosis reduction, but again, the underlying mechanism between foam cell formation and phagocytosis reduction remains unclear.

Macrophages are phagocytes that are meant to remove pathogens as well as damaged or dead cells. Macrophages take up the lipoproteins that are acquired from the phagocytosis of dying cells; hence, they have developed mechanisms cholesterol elimination. If excess cholesterol is not eliminated from the macrophages, foam cell transformation might occur.

During macrophage phagocytosis, lipids are degraded in the lysosome by lysosomal acid lipase into free cholesterol and fatty acids [11]. In the cytosol, fatty acids are activated to acyl-coenzyme A (Acyl-CoA) either for lipid biosynthesis or for mitochondrial fatty acid β-oxidation (FAO) [12]. The enzyme carnitine palmitoyltransferase-1 (CPT1) converts Acyl-CoA to acylcarnitine. This complex is then translocated across the inner mitochondrial membrane to the mitochondrial matrix by the carnitine–acylcarnitine translocase and finally reconverted back to Acyl-CoA by the enzyme CPT2 of the peripheral inner mitochondrial membrane [13].

Increasing FAO in lipid-laden foam cells, such as those observed in atherosclerosis, by enforcing CPT1a expression can reduce lipid accumulation as well as the production of pro-inflammatory cytokines [14], suggesting that inducing FAO in foam cells could be of therapeutic potential. Thus, CPT1a expression seems to be related to foam cell formation, and previous results indicate that the induction of macrophage foam cell formation, phagocytosis, and inflammatory phenotype are dependent on intracellular lipid accumulation and CPT1a expression, and that the downregulation of CPT1a by high lipid content in the macrophages is a key modulator of this process [15]. Additionally, macrophages internalize low-density lipoproteins (LDLs) [16] that are degraded through lysosomal lipolysis into free cholesterol and fatty acids, and these are then exported to high-density lipoproteins (HDLs) or transported into the mitochondria, respectively. Fatty acids are transported to the mitochondria via the carnitine shuttle system for energy production through FAO, in which CPT1a is considered to be the rate-limiting enzyme in FAO [17].

Since CPT1a is a mitochondrial enzyme that is downregulated in cholesterol damaged cells, we think that its enrichment in healthy mitochondria by mitochondrial transfer could restore the alterations in CPT1a expression and, thus, the phagocytic ability of the macrophages.

The present study aims to prove that the transplantation of healthy mitochondria to highly cholesterol loaded macrophages, restores the lost phagocytic function and reduces macrophage shift towards foam cells.

## 2. Materials and Methods

### 2.1. Materials

Dulbecco’s Modified Eagle Medium/Nutrient Mixture F-12 with GlutaMAX (DMEM/F12), Heat Inactivated Fetal Bovine Serum (FBS), and Penicillin/Streptomycin were obtained from Gibco (Madrid, Spain). Live cell image solution and pHrodo Green E. coli BioParticles conjugate were purchased from Molecular Probes (Madrid, Spain). MitoTracker Red CMXRos and MitoTracker Green FM were obtained from Invitrogen (Thermo Fisher, Waltham, MA, USA). The compound 7-Ketocholesterol (5-Cholesten-3β-ol-7-one) was purchased from Santa Cruz Biotechnology, Inc (Heidelberg, Germany). The 7-ketocholesterol stock solution was prepared by dissolving the chemical compound in 100% ethanol at a concentration of 5 mg/mL. The designed primers were bought from Bio-Rad and Invitrogen. Alamar Blue reagent (Thermo Fisher, Waltham, MA, USA) was added (10 μL Alamar Blue per 100 μL sample) and incubated for 4 h at 37 °C. Colour changes and increased fluorescence were quantified using absorbance at the respective excitation wavelength of 570 and 600 nm.

### 2.2. Cell Culture

The murine macrophage cell line RAW264.7 (obtained from the European Collection of Authenticated Cell Cultures) was cultured in DMEM/F12 supplemented with 10% FBS and 1% antibiotics (100 Units/mL penicillin and 100 μg/mL streptomycin). Cells were maintained in a humidified incubator at 37 °C under 5% CO2 and passage before reaching 80% confluence by cell scraping. Cells from passages 10–15 were seeded in a 12-well plate at a cell density of 2.5 × 10^5^ per well and were allowed to grow for 24 h. Unless otherwise stated, each sample had at least an *n* = 3, and each experiment was reproduced at different times.

### 2.3. Oil Red O Staining (ORO)

To prepare the ORO working solution, three parts 0.3% ORO was mixed with two parts distillated water, allowed to settle for 10 min, and filtered before use. Cells were fixed with 10% formalin for 10 min, washed with PBS, rinsed with 60% isopropanol for 5 min, and stained with ORO working solution for 5 min. Nuclei were counterstained with hematoxylin. Images were acquired in a Zeiss Axiophot microscope. For ORO staining, quantification cells were transferred to a 96-well plate, and the absorbance was read at 510 nm in a Multiskan Sky Microplate Spectrophotometer (Thermo Fisher Scientific, Waltham, MA, USA).

### 2.4. Phagocytosis Assay

One vial of pHrodo Green E. coli BioParticles conjugate (P35366) was suspended in 2 mL of LCIS (Live Cell Imaging Solution, A14291DJ) at 1 mg/mL and was thoroughly vortexed and sonicated following manufacturer’s instructions. Bioparticles were titered to a final concentration of 55 μg/mL per well for the assay. After each experiment, RAW264.7 cells were incubated with pHrodo Green E. coli BioParticles diluted in LCIS at 55 μg/mL for 90 min at 37 °C. Cell imaging was performed on a Leica CTR 4000 microscope and fluorescence intensity was measured using a Spectramax Gemini XS spectrofluorometer plate reader (Molecular Devices, Sunnyvale, CA, USA) at an excitation of 485 nm and an emission of 530 nm. As indicated by the manufacturers, net phagocytosis was calculated by subtracting the average fluorescence intensity of the no-cell negative control wells from all sample wells. Net phagocytosis data are presented as the change level relative to that observed in the untreated control cells.

### 2.5. Quantitative Real Time Polymerase Reaction (qPCR)

Total RNA was isolated using the RNeasy Mini kit (Qiagen, Hilden, Germany). Reverse transcription was carried out with 1 μg of total RNA through the iScript cDNA synthesis Kit (Bio-Rad, Hercules, CA, USA). qPCR assay was preformed using the SsoAdvanced Universal SYBR Green Supermix on a 96-well CFX96 Touch Real Time PCR detection system from Bio-Rad. Primers sequences are listed in Table 1. The commercial primers acquired from Bio-Rad are listed in Table 2. Technical replicates were performed in each sample, and relative mRNA expression was calculated using the 2^−^^ΔΔCT^ method. The results that were obtained represent the fold changes in the target gene in the test sample relative to the control sample comprising the untreated cells. Each sample was previously normalized to the expression of the reference gene 18s RNA.

### 2.6. Isolation of Mitochondrial Fraction

Mitochondria were isolated using the Mitochondria Isolation Kit for culture cells (Thermo Fisher Scientific) following provider instructions. In brief, 2 × 10^7^ RAW264.7 cells were homogenized, centrifuged at 1.100 g for 18 min, and the supernatant was centrifuged at 12.000 g for 15 min at 4 °C to pellet the mitochondria. The mitochondria pellets were re-suspended in the mitochondrial extraction buffer from the kit and proportioned in the kit and centrifuged at 20.000 g for 10 min at 4 °C. Following for a second centrifugation at 20.000 g for 5 min at 4 °C, the final pellet re-suspended in PBS. The isolated mitochondria were quantified by determining the protein concentration using the Bio-Rad Protein Assay. To test active isolated mitochondria, we used a MitoTracker Red CMXRos probe that stains mitochondria, and its accumulation is dependent on the mitochondrial membrane potential. In addition to the evaluation of Red CMXROS viability, in order to further confirm the viability of the mitochondria, we assayed the oxygen uptake of the suspension following Simon et al. [18] with a Clark-type chamber at 37 °C. The mitochondrial pellet was resuspended in a respiratory buffer containing 5 mM HEPES, 220 mM manitol, 70 mM sucrose, 1 mM KH2PO4, 5 mM succinate, 0.1 mM ethylenediaminetetraacetic acid, and 0.1% bovine serum albumin (pH 7.2). Briefly, oxygen consumption was checked as follows: 200 mM adenosine diphosphate was added to 225 microliters of a mitochondria suspension in 2.5 mL of respiratory buffer to initiate the viability register.

### 2.7. Mitochondrial Transfer

Prior to mitochondrial transfer, recipient cells were labelled with MitoTracker Green FM to mark the endogenous mitochondria. Cells were harvested from culture flasks, and 1 × 10^5^ cells were transferred to a microcentrifuge tube. Cells were resuspended in 100 μL of PBS and kept on ice for transfer. After that, 10 μg of isolated mitochondria previously labelled with MitoTracker Red CMXRos was slowly added to each tube of recipient cells suspended in 100 μL of PBS. The microcentrifuge tubes were centrifuged at 1.500 g for 5 min at 4 °C, rinsed twice with PBS, and imaged or lysed for further testing. The transfer was confirmed by image analysis and electron microscopy. The mitochondria that had been isolated from RAW264.7 cells were transplanted into 7-ketocholesterol-loaded cells (incubated with 10 μg/mL of 7-KC in DMEN for 24 h) or exposed to absolute ethanol as a vehicle. The viability of the cell culture was checked using the Alamar Blue viability assay. According to the collected data, cell viability was above 95% for all of the treatments.

### 2.8. Electron Microscopy

After 2 h of mitochondrial transplantation and mitochondria isolation, the mitochondria were fixed with glutaraldehyde 2.5% and paraformaldehyde 2% in buffer phosphate (0.1 M, pH 7.4), post-fixed in 1% osmium tetroxide and 0.8% potassium ferrocyanide, dehydrated with acetone, and embedded in epoxy resin. Sections were cut and stained with methyleneblue for light microscopy. Ultrathin sections for transmission electron microscopy were cut and stained with 2% uranyl acetate for 10 min and with a lead-staining solution for 2 min. Images from stained ultrathin sections were acquired by moving randomly across the EM grid using a transmission electron microscope JEOL JEM-1010 fitted with a Gatan Orius SC1000 (model832) digital camera.

### 2.9. Statistical Analysis

All data were reported as mean  ±  SEM of at least three independent experiments, and each experiment had an *n*  =  4. The means of each group were compared with One-way analysis of variance (Anova) followed by Tukey’s post hoc test for multiple comparisons. Values were considered statistically significant if the value of *p* <  0.05. Statistical analyses were performed with GraphPad Prism 9.0 software.

## 3. Results

### 3.1. Efficiently Transferred Mitochondria between Macrophages

**Figure 1:**
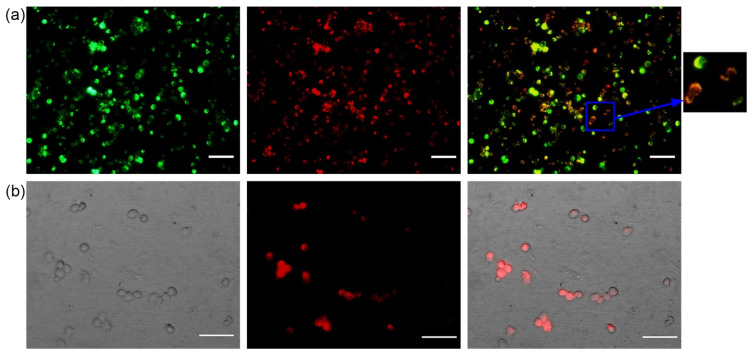
Microscopic analysis of target cells following mitochondrial transfer. (**a**) Representative images of mitochondrial transplantation between macrophages co-stained with fluorescent mitochondrial dyes (MitoTracker Green and MitoTracker Red CMXRos). Green: endogenous mitochondria of recipient cells, red: transferred mitochondria isolated from macrophages, yellow: merged mitochondria. Scale bar 50 μm (magnification ×20). (**b**) Fluorescence microscopy of recipient cells after mitochondrial transfer. The last image on the right corresponds to the merge image of staining exogenous mitochondrial versus visible microscopy. Scale bar 50 μm (magnification ×40). Representative pictures of six independent experiments are shown. Cells were viewed on a Leica TCS NT laser microscope.

Exogenous mitochondria stained with MitoTracker Red CMXRos were mixed with macrophages whose endogenous mitochondria were stained with MitoTracker green FM and then immediately subjected to centrifugation. As shown in Figure 1a, exogenous mitochondria were transferred between macrophages, as evidenced by the yellow color in the merged image when the transferred exogenous mitochondria (red) co-localized with the endogenous mitochondria (green). Our results clearly show the presence of the transferred mitochondria within the recipient cells, confirming that exogenous mitochondria can be efficiently transferred into recipient cells by centrifugation.

As shown in Figure 1b, the mitochondria were incorporated after transplantation. Therefore, once it has been verified that the process was working properly, the mitochondrial extraction experiment was performed with 20 million RAW264.7 cells and transferred to macrophages that had been previously loaded with 7-ketocholesterol (7-KC).

**Figure 2:**
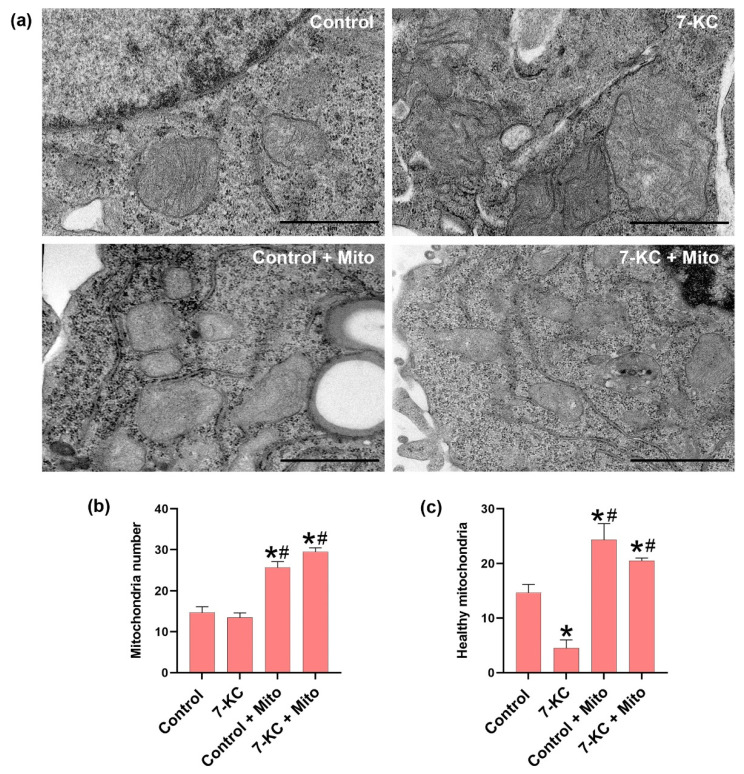
Electron microscopy analyses of control 7-ketocholesterol-loaded macrophages with (7-KC + Mito) or without (7-KC) mitochondrial transplantation. (**a**) Representative images acquired with a Gatan Orius digital camera by moving randomly across the EM grid and are representative of three replicates per group. (**b**) Mitochondrial number quantified from images of ultrathin sections and analyzed using ImageJ software. (**c**) Number of healthy mitochondria quantified from images of ultrathin sections and analyzed using Image J software. Data are presented as means ± SEM, *n* = 3, *p* ≤ 0.05 * vs. control, # vs. 7-KC loaded cells.

Figure 2 shows that the mitochondria are altered in the 7-KC-loaded macrophages, while in the controls, the structure is maintained. Electron microscopy analyses revealed that the mitochondria from 7-KC loaded macrophages appeared with abnormal mitochondrial ridges compared to mitochondria from the controls. These findings indicate that cholesterol traffics to the mitochondria and alters the mitochondrial morphology.

An increased number of mitochondria was detected after mitochondria transplantation to the control macrophages and to the 7-KC-loaded macrophages. However, in the control macrophages with mitochondrial transplantation, the structure of the mitochondria was normal in contrast to the 7-KC-loaded macrophages that had been transplanted with mitochondria, allowing us to see both normal and altered mitochondria. Nevertheless, a high number of healthy mitochondria could be observed after mitochondrial transplantation in both groups, indicating that the transplantation of mitochondria is effective in increasing the number of mitochondria in the recipient cells.

### 3.2. Mitochondrial Transfer between Macrophages Shifts Macrohage Phenotype

**Figure 3:**
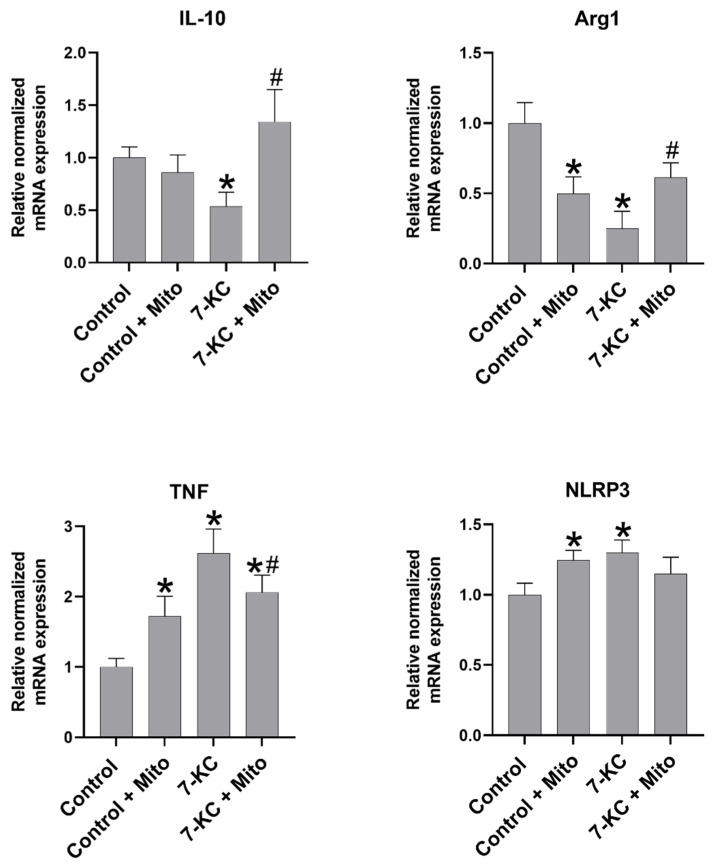
Healthy mitochondrial transfer between macrophages is involved in macrophage phenotype switch. mRNA expression determined by qPCR of TNF, arginine, NLRP3, and IL10. Data are represented as means ± SEM of six independent experiments with *n* = 3 each. *p* ≤ 0.05 * vs. control, # vs. 7-KC-loaded cells.

As observed in Figure 3, the cells loaded with 7-KC express a pro-inflammatory phenotype that was changed after mitochondrial transplantation. The 7-KC-loaded cells expressed a high increase in TNF expression and NLRP3, which were reduced when they received healthy mitochondrial transplantation. In the same sense, the 7-KC-loaded cells expressed a significant decrease in arginine and IL-10 expression that was increased when the mitochondria were transferred. Thus, this indicates the effect of mitochondrial transfer in changing the macrophage phenotype. In contrast, the mitochondrial transfer to the control macrophages promoted a significant increase in TNF and NLRP3 expression and a significant decrease in arginine expression when compared to the control macrophages.

### 3.3. Mitochondrial Tranfer between Macrophages Modulates Cell Lipid Content, Phagocytosis and CPT1a Expression

**Figure 4:**
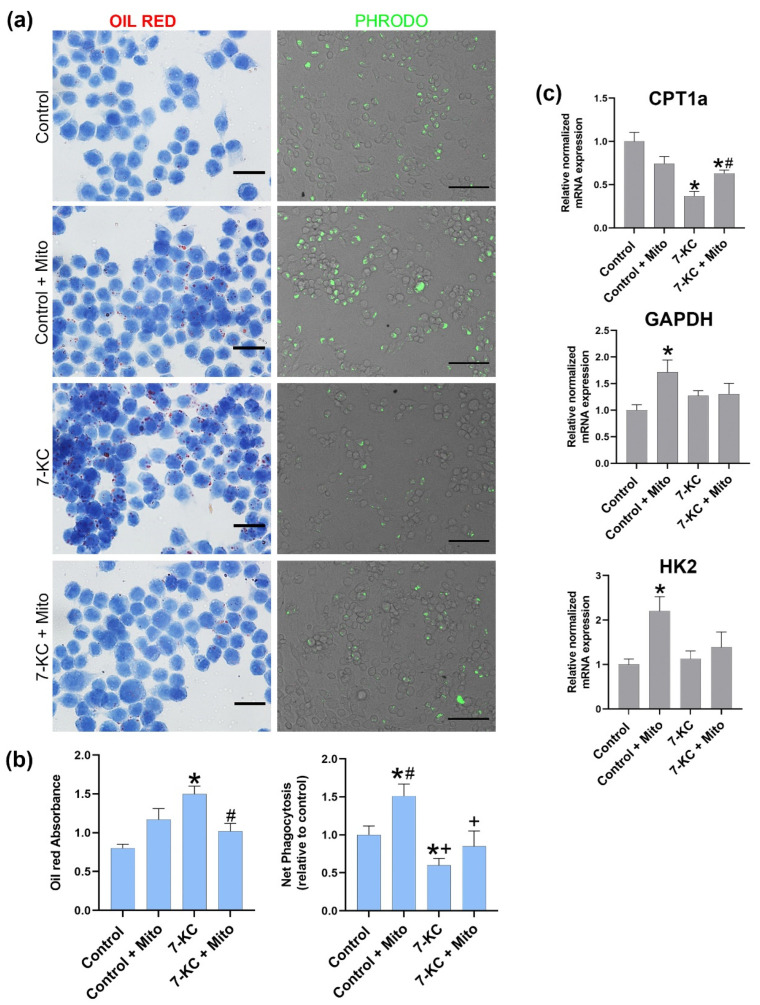
Effects of mitochondrial transfer on macrophage lipid content, mRNA expression CPT1a, GAPDH, HK2, and phagocytosis. RAW264.7 were incubated with 7-KC at 10 μg/mL for 24 h. As a control, the cells were incubated with vehicle ethanol for 24 h. (**a**) Representative Oil Red O staining images, scale bar 20 μm. Representative images of pHrodo uptake capture by fluorescence microscopy, scale bar 20 μm.(**b**) Oil red Absorbance at 510 nm and Net Phagocytosis of 55 μg/mL pHrodo Green E. ecoli bioparticles conjugate for 90 min measured by spectrofluorometer at excitation 485 nm and emission 530 nm. (**c**) qPCR analysis of mRNA expression levels of CPT1a, HK2, and GAPDH represented relative to the control of the experiments. Data are represented as means ± SEM of 3 independent experiments with *n* = 3 each. *p* ≤ 0.05 * vs. control; # vs. 7-KC, + vs. control + mitochondria.

As observed in Figure 4, cells exposed to 7-KC showed a reduced expression of CPT1a that was significantly increased after mitochondrial transplantation. Consistently, these results are in line with the increase in oil red staining in the 7-KC-loaded cells that was significantly reduced when the mitochondria were transplanted. In addition, phagocytosis was decreased in these 7-KC-treated macrophages but was recovered after mitochondrial transplantation, thus indicating the ability to revert foam cell formation after mitochondrial transplantation in lipid-laden macrophages.

On the contrary, mitochondrial transfer to the control macrophages did not significantly modify CPT1a expression and oil red staining while phagocytosis increased significantly. GADPH and HK2 expression were significantly increased after mitochondrial transplantation to control the macrophages, while it remained unmodified after mitochondrial transplantation in cholesterol-loaded macrophages, indicating that mitochondrial transplantation only affects lipolytic enzymes and not glycolytic ones.

## 4. Discussion

The compound 7-KC is one of the most abundant oxysterols in oxLDL [19] and is found in and atherosclerotic plaques [20], and we used it to induce macrophages with a high lipid load. We have treated RAW264.7 cells with 7-KC and examined the alterations in oil red accumulation and CPT1a gene expression. As shown in Figure 4, 7-Keto cholesterol treatment induced an increase in lipid-load charge and a decrease in CPT1a expression, confirming the effect of 7-KC on the altered metabolism and in foam cell formation.

Previously, 7-KC has been shown to cause mitochondrial dysfunction in cultured cells, namely a loss in mitochondrial membrane potential that leads to reduced oxidative phosphorylation and reduced ATP production [21]. It is known that the increase in cholesterol in mitochondrial membranes changes the physical membrane properties [22,23] and that the loss of mitochondrial fluidity due to cholesterol enrichment altered the mitochondrial morphology. It has been reported that the electron microscopy analyses of mitochondria from cholesterol-treated mice appeared rounded and with abnormal levels of cristae compared to the mitochondria from the control animals [23]. Our results confirm that cholesterol damages mitochondria, as shown in Figure 2. Mitochondria that have been damaged by 7-KC could alter the effectivity of the mitochondrial enzyme CPT1a, showing a decreased expression after 7-KC treatment (Figure 4), thus leading to an increase in the lipid load and deficient phagocytosis.

It is known that mitochondrial transfer can take place between MSCs and immune cells, which influences the functions/properties of the immune cells [24]. Using an acute respiratory distress syndrome (ARDS) model, it was found that MSCs can donate mitochondria to host macrophages and enhance the phagocytic capacity and bioenergetics of macrophages, leading to an improved clearance of pathogenic bacteria [25,26]. However, the factors that contribute to the improved phagocytic capacity while showing a reduced proinflammatory reaction after mitochondrial transfer remains elusive.

Our previous studies have shown that CPT1a expression is related to phagocytosis since silencing CPT1a reduces phagocytosis as a consequence of the increased lipid load, while overexpressing CPT1a increases phagocytosis and reduced lipid load [15]. As observed in Figure 4, mitochondrial transplantation to 7-KC-loaded cell macrophages is effective since healthier mitochondria can be detected and increase CPT1a expression, reduce oil red charge, and increase phagocytosis, thus indicating that mitochondrial transplantation trough increased CPT1a expression could modulates phagocytosis and foam cell formation.

It could be argued that the mitochondrial increase after mitochondrial transplantation to 7-KC-loaded cells is probably the cause of the increased CPT1a expression with its consequences in FAO improvement and thus the reduction in the lipid-load charge that results in an increase in the phagocytic potential of the cells.

Other authors have found that mitochondrial transfer is associated with a change towards the macrophage M2 phenotype. Using an ARDS model, Morrison et al. reported that the extracellular vesicle-mediated transfer of mitochondria can induce monocyte-derived macrophages (MDMs) to differentiate macrophages to an M2 phenotype with high phagocytic capacity, and this phenotypic change was mediated by mitochondrial transfer that requires the OXPHOS process to be present in the macrophages [24]. In another study, Kim and Hematti co-cultured MSCs with macrophages in vitro and found that MSCs can educate macrophages to adopt an IL-10-high, IL-12-low (Interleukin 12-low), IL-6-high (Interleukin 6-high), and low-TNF phenotype, an anti-inflammatory phenotype similar to the M2 one [27]. Our results are in line with these previous observations since, as shown in Figure 3, mitochondrial transplantation to cholesterol-treated cells restored the M2 phenotype since IL10 and arginine expression are significantly increased with respect to the reduced levels observed in 7-KC macrophages while NLRP3 and TNF decreases with respect to 7-kc treated cells. Indicating the restoration of M2 phenotype after mitochondrial transplantation.

As such, a correlation between the stimulation of CPT1a expression and a switch in the M1 phenotype towards M2 was found in 7-KC-treated cells. This result is in line with other authors who found that the expression of a malonyl-CoA-insensitive form of CPT1A on RAW264.7 macrophages not only enhanced FAO but also reduced the production of proinflammatory cytokines from palmitate-induction [14].

However, a change in the macrophage phenotype was observed when the mitochondria were transplanted into the control macrophages. As shown in Figure 3, a significant increase in NLP3 and TNF was observed when the mitochondria were transplanted to the control macrophages, thus indicating that mitochondrial transplantation was only effective when the cells were altered by 7-KC. The fact that the transfer of the mitochondria to healthy and 7-KC-loaded macrophages seems to have opposing effects on gene expression could be attributed to the differential effects on glycolytic genes.

In this sense, it is known that cellular metabolism also contributes to macrophage activation, as it has been noticed that the M1 macrophages rely on glycolysis, whereas the M2 macrophages seem to prefer fatty acid oxidation and glutamine metabolism as an energy source [28,29,30].

The energy metabolism in M1-polarized macrophages shifts to glycolysis compared to their precursors, and the M1-polarized macrophages are activated by mtDNA and mitochondria-produced ROS. Energy metabolism in M2-polarized macrophages mainly depends on mitochondrial respiration fueled by oxygen and fatty acid bacteria [31].

Since we also assessed the effect of mitochondrial transplantation in glycolysis-associated genes and, as observed in Figure 4, mitochondrial transplantation to control macrophages produced an increase in GAPDH and HK2, this indicates that mitochondrial transplantation initiates metabolic reprogramming in tissue macrophages toward glycolysis and has the potential to promote pro-inflammatory activity, which can be observed when the mitochondria were transplanted to control the macrophages.

In contrast, glycolysis-associated genes were unmodified by mitochondrial transplantation in cholesterol treated macrophages, indicating that mitochondrial transplantation to 7-KC-loaded cells does not modify metabolic reprogramming in tissue macrophages toward glycolysis and the M1 phenotype, but modified it towards the FAO and M2 phenotype.

In summary our results indicate that mitochondrial transplantation to cholesterol-damaged cells induces CPT1a expression and changes the macrophage phenotype. In addition, the lipid load is reduced, leading to an increase in the phagocytic ability. Thus, mitochondrial transplantation between macrophages could represent a new therapeutic tool to improve the damaging consequences of lipid-loaded macrophages.

## Figures and Tables

**Table 1:** Sequence of primers from Invitrogen.

Genes	Forward	Reverse	Efficiency (%)
18s RNA	CCTGCGGCTTAATTTGACTC	GACAAATCGCTCCACCAACT	97
Arg 1	AGACCACAGTCTGGCAGTTG	TGTCAGTGTGAGCATCCACC	102

18s RNA: 18s ribosomal RNA; Arg 1: arginine 1.

**Table 2:** Primer-unique assay identification (ID) number and chromosome location of PrimePCR™ SYBR^®^ Green Assay by Bio-Rad Laboratories.

Genes	Unique Assay ID	Chromosome Location	Efficiency (%)
TNF	qMmuCED0004141	17:35201717-35201865	98
CPT1a	qMmuCED0045595	19:3385535-3385661	98
IL-10	qMmuCED0044967	1:131024363-131024476	100
HK2	qMmuCED0045344	6:82749208-82749307	101
GAPDH	qMmuCED0027497	6:125162278-125162382	101
NLRP3	qMmuCID0010647	11:59558525-59565111	101

TNF: tumor necrosis factor; CPT1a: carnitine palmitoyltransferase-1a; IL-10: interleukin 10; HK2: hexokinase 2; GAPDH: glyceraldehyde 3-phosphate dehydrogenase; NLRP3: NOD-like receptor pyrin domain-containing-3.
